# Outcomes of Conservative and Surgical Management of Trigger Thumb in Children Up to Five Years at a Tertiary Care Center in Kolkata

**DOI:** 10.7759/cureus.104993

**Published:** 2026-03-10

**Authors:** Pradyumna Chakraverty, Malay K Mandal, Sourav Das, Nilay K Das, Arnab Sarkar, Madhumita Bhakta

**Affiliations:** 1 Department of Orthopedics, KPC Medical College and Hospital, Kolkata, IND; 2 Department of Orthopedics, West Bengal Health Services, Kolkata, IND; 3 Department of Health and Family Welfare, Government of West Bengal, Kolkata, IND; 4 Department of Community Medicine, Saheed Laxman Nayak (SLN) Medical College and Hospital, Koraput, IND; 5 Department of Community Medicine, Maharaja Krishna Chandra Gajapati (MKCG) Medical College and Hospital, Berhampur, IND

**Keywords:** child, preschool, tenosynovitis, thumb, trigger finger disorder

## Abstract

Introduction: Pediatric trigger thumb is a developmental condition characterized by flexion deformity of the interphalangeal joint due to constriction at the A1 pulley. Although spontaneous resolution has been reported, optimal treatment timing remains controversial. This study aimed to compare outcomes between conservative and surgical management in children aged up to five years.

Methodology: A prospective analytical cohort study was conducted at a tertiary care center in Kolkata over 18 months. Fifty-eight children with clinically diagnosed trigger thumb were enrolled. All participants initially underwent six months of conservative observation. Those who did not improve underwent open A1 pulley release. The primary outcome was complete resolution of deformity. Secondary outcomes included degree of improvement, recurrence, complications, and time to resolution. Statistical analyses included the chi-square test, the Mann-Whitney U test, the Cox proportional hazards model, and multivariable logistic regression.

Results: Nineteen children (32.8%) improved with conservative management, whereas 39 (67.2%) required surgery. Surgical intervention achieved 100% resolution with no recurrence or major complications. Mean improvement in deformity was significantly greater in the surgical group (50.8° ± 5.8°) compared with the conservative group (14.5° ± 3.7°; p < 0.001). The relative risk of complete resolution with surgery was 3.05 (95% confidence interval (CI): 2.05-4.55), with an absolute risk reduction of 67.2% and a number needed to treat of approximately two. Cox analysis demonstrated faster recovery with surgery (HR: 4.62; 95% CI: 2.71-7.88; p < 0.001). An initial deformity of >20° independently predicted conservative failure.

Conclusion: Surgical release provides significantly superior and faster resolution compared to conservative management, particularly in children presenting with deformity greater than 20°.

## Introduction

Pediatric trigger thumb is a developmental condition rather than a true congenital anomaly, typically presenting between ages one and three rather than at birth [1,2]. Clinically, it is characterized by flexion deformity of the interphalangeal (IP) joint of the thumb, inability to achieve active extension, and a palpable nodule at the level of the first annular pulley (A1 pulley), commonly referred to as Notta’s nodule [3]. The underlying pathology involves a size mismatch between the flexor pollicis longus tendon and the A1 pulley, leading to mechanical constriction and tendon entrapment without the inflammatory component observed in adult trigger digits [2,4]. Bilateral involvement has been reported in approximately 25%-30% of cases [1,5]. The reported incidence ranges from one per 2,000 live births to three per 1,000 children [1,5]. Although uncommon, the condition carries functional and cosmetic implications, particularly when deformity becomes fixed. Management remains controversial and broadly includes conservative strategies, observation, splinting, or passive stretching, and surgical release of the A1 pulley.

The natural history of pediatric trigger thumb has been widely investigated. Long-term observational studies have reported spontaneous resolution rates of 60%-75% over extended follow-up periods [6,7]. Resolution appears more likely in milder or triggering-type deformities, while locked thumbs demonstrate lower rates of spontaneous recovery [8]. Splint therapy has demonstrated variable success, with reported improvement rates ranging from approximately 55% to over 90% depending on protocol and compliance [9,10]. However, conservative approaches require prolonged follow-up and may not be effective in more severe deformities.

Open surgical release of the A1 pulley remains the most definitive treatment in children with persistent, fixed, or severe pediatric trigger thumb that fails to improve with an adequate trial of conservative management, particularly when the deformity is fixed (locked) in flexion, initial flexion deformity is greater than 20°, there is no improvement after three to six months of observation, functional limitation is present, the child is older (e.g., greater than two to three years) with established contracture, and there is parental preference for definitive correction. It is especially indicated when spontaneous resolution is unlikely, such as in locked thumbs or cases with progressive deformity. Multiple series have reported success rates approaching 95%-100% with low recurrence and minimal complications [11-13]. Comparative analyses have demonstrated superior outcomes with open release compared with splinting or passive exercises [14]. Concerns have been raised regarding percutaneous techniques in children because of potential neurovascular or tendon injury; therefore, open release is generally recommended in the pediatric population [15,16].

Despite the large body of literature, several limitations persist. Many studies are retrospective, use heterogeneous inclusion criteria, and apply variable follow-up durations. Conservative protocols differ significantly in splint type, duration, and compliance monitoring. Moreover, most published data originate from Western or East Asian cohorts, with limited prospective evidence from Indian tertiary-care centers. Socioeconomic factors, access to care, and adherence to follow-up may influence outcomes in this context. A structured prospective comparison within a local population is therefore warranted. Given the reported spontaneous resolution rates of 60%-75% over long-term follow-up [6-8] and the consistently high success rates of surgical release (95%-100%) [11-14], there remains uncertainty regarding the optimal timing of intervention, particularly in children under five years of age.

Objectives

The primary objective of this study was to compare outcomes between conservative and surgical management of pediatric trigger thumb in children aged up to five years. Secondary objectives included evaluating resolution rates, time to full IP extension, recurrence, and complications, and identifying clinical predictors of treatment outcomes.

## Materials and methods

This prospective analytical cohort study was conducted to compare the outcomes of conservative and surgical management of pediatric trigger thumb in children aged five years or younger. The study was carried out in the Outpatient Department and Department of Orthopedics at the KPC Medical College and Hospital, Kolkata, a tertiary-care referral center serving both urban and rural populations. The study period extended over 18 months, from January 2024 to June 2025, following approval from the Institutional Ethics Committee. Written informed consent was obtained from the parents or legal guardians of all participants prior to enrollment.

All eligible children presenting during the study period were enrolled consecutively and followed prospectively. Children aged zero to five years with clinically diagnosed pediatric trigger thumb and symptom duration of at least six weeks were included. Diagnosis was made clinically based on the presence of flexion deformity at the IP joint of the thumb, with or without a palpable Notta’s nodule. Children older than five years, those who had undergone previous trigger thumb surgery, those who had received conservative treatment for more than six months prior to presentation, those with associated congenital hand anomalies or systemic conditions affecting hand function, children medically unfit for surgery, or those unlikely to comply with follow-up were excluded. Symptoms suggesting milder disease and, therefore, a higher probability of response to observation included intermittent triggering, the ability to actively or passively extend the thumb, absence of a fixed flexion contracture, and minimal functional limitation. These children were generally managed conservatively during the observation period. In contrast, symptoms indicating more advanced disease included persistent or fixed flexion deformity (locked thumb), inability to actively extend the IP joint, progressive worsening of deformity, and functional impairment during grasping or fine motor activities. The presence of a prominent Notta’s nodule and a higher degree of flexion deformity (>20°) also suggested a lower likelihood of spontaneous resolution. Children with these features were more likely to require surgical release if no improvement was observed during follow-up.

The sample size was calculated using the formula for comparing two proportions, assuming a 63% success rate for conservative management and 95% for surgical management, with a 95% confidence level and an allowable error of 20%. The minimum calculated sample size was 54 participants. To account for possible attrition and ensure adequate statistical power, 58 children were ultimately enrolled.

At baseline, a detailed clinical evaluation was performed. Demographic data, including age and sex, were recorded, along with laterality (unilateral or bilateral involvement), progression history, and the presence of Notta’s nodule. The degree of flexion deformity at the IP joint was measured using a standard goniometer to ensure objective quantification. For children with bilateral involvement, analysis was performed per patient rather than per thumb. In such cases, the thumb with greater initial deformity was considered for primary outcome analysis, and resolution required correction in both thumbs.

All participants initially underwent conservative management consisting of observation alone for six months, without splinting, structured passive stretching, or activity restriction. Splinting was not included in the conservative management protocol to maintain a standardized and easily reproducible observation-based approach for all participants. In young children, prolonged splint use is often associated with variable compliance and inconsistent application by caregivers, which can affect treatment outcomes. Additionally, previous studies have reported heterogeneous success rates with splint therapy due to differences in splint type, duration of use, and adherence. To minimize these confounding factors and ensure uniform treatment exposure across the study cohort, conservative management in this study was limited to observation during the initial follow-up period. At the end of this observation period, outcomes were categorized as complete response (achievement of neutral or full active IP extension), partial response (≥50% reduction in baseline deformity without progression), or nonresponse (<50% improvement, persistent fixed deformity, or worsening). Children demonstrating a complete response continued observation. Partial responders were monitored further unless deformity plateaued or progressed. Children classified as nonresponders were offered surgical intervention after shared decision-making with parents. This represented a staged sequential treatment pathway.

Surgical management consisted of open A1 pulley release performed under general anesthesia with tourniquet control in the institutional operating theatre. A transverse incision was made at the volar metacarpophalangeal crease of the thumb. The digital nerves were carefully identified and protected. The A1 pulley was incised longitudinally under direct visualization, and complete release was confirmed intraoperatively by restoration of full IP joint motion. The wound was closed and dressed without postoperative immobilization. Gentle passive thumb movements were initiated on the first postoperative day. Surgically treated children were followed for an additional six months to monitor resolution, recurrence, and complications.

The primary outcome measure was resolution of flexion deformity, defined as achievement of neutral or full IP extension. Secondary outcomes included the magnitude of deformity improvement, recurrence rate, and incidence of complications. Data were collected using a structured clinical proforma and entered into Microsoft Excel (version 2019; Microsoft Corporation, Redmond, WA) for analysis using JAMOVI version 2.6.44 (Computer Software. Retrieved from https://www.jamovi.org) and R Studio version 4.5.2 (Posit Software, Boston, MA). Quantitative variables were expressed as mean with standard deviation and median with interquartile range, while categorical variables were presented as frequencies and percentages. Normality was assessed using the Kolmogorov-Smirnov test. Comparisons between treatment groups were conducted using Pearson’s chi-square test for categorical variables when expected cell counts were adequate; Fisher’s exact test was applied when expected counts were less than five. For continuous variables not meeting normality assumptions, the Mann-Whitney U test was used. For the Mann-Whitney analysis, the U statistic and corresponding standardized Z value were calculated and reported. Descriptive effect measures were calculated to compare resolution rates between the groups, including relative risk (RR), absolute risk reduction (ARR), and number needed to treat (NNT), along with 95% confidence intervals (CIs). Given the nonrandomized design and treatment allocation based on clinical response, these measures represent associative estimates rather than causal effects and were interpreted cautiously.

Time-to-resolution analysis was conducted using Cox proportional hazards regression with surgery treated as a time-dependent covariate to minimize immortal time bias. The primary event was defined as the achievement of a neutral or fully active IP extension. Time was measured in months. For all participants, the time origin was defined uniformly as the date of study enrollment (baseline evaluation). This ensured a common starting point for both treatment groups.

Definition of time-to-resolution

Conservative Group

Time-to-resolution was defined as the interval from enrollment to the first documented visit at which complete resolution (neutral or full active IP extension) was achieved during the observation period.

Surgical Group

Surgery occurred after the initial six-month observation period for nonresponders. To avoid altering the time origin, time-to-resolution in the surgical group was defined as the interval from enrollment (not from surgery date) to the first postoperative visit demonstrating complete correction.

Before the date of surgery, patients contributed person-time to the conservative exposure category. After surgery, the exposure status switched to the surgical category. This time-dependent approach ensured that no presurgical “immortal” time is incorrectly attributed to the surgical group.

Participants were censored under the following conditions.

Conservative Group

1) Censored at six months if no resolution occurred and the child proceeded to surgery.

2) Censored at last follow-up if lost to follow-up without documented resolution.

Surgical Group

1)** **Censored at the last postoperative follow-up if no recurrence occurred.

2) Censored at time of loss to follow-up, if applicable.

No participants were censored after experiencing the primary event.

Multivariable logistic regression analysis was performed to identify independent predictors of resolution. A p value of less than 0.05 was considered statistically significant. Quality assurance was ensured through standardized measurement techniques and uniform surgical protocols performed by experienced orthopedic surgeons. Confidentiality was maintained throughout the study, and participants were free to withdraw at any time without affecting their treatment.

## Results

Participant characteristics

**Figure 1:**
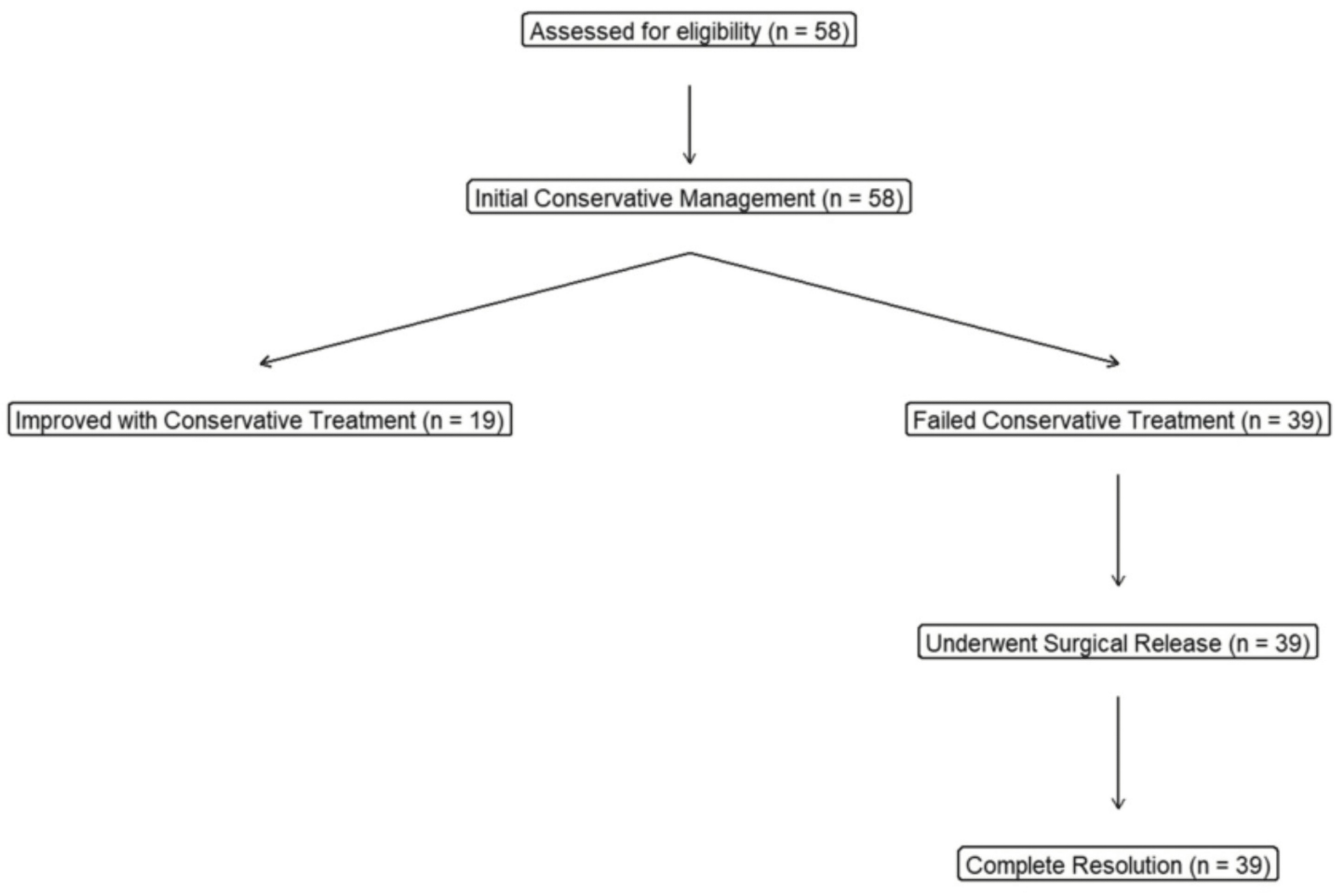
Flow diagram of participant recruitment, allocation, and follow-up in the prospective cohort study

A total of 58 children aged less than or equal to five years with clinically diagnosed pediatric trigger thumb were enrolled. All participants initially received conservative management for six months. Nineteen children (32.8%) improved and required no further intervention, while 39 children (67.2%) showed no improvement and subsequently underwent open A1 pulley release (Figure 1).

The mean age at presentation was 23.97 ± 13.1 months (median 24 months). Sex distribution was equal (29 male and 29 female participants). Unilateral involvement was present in 72.4%, and bilateral involvement in 27.6%. Notta’s nodule was identified in 93.1% of cases. Baseline characteristics are summarized in Table 1. The mean initial flexion deformity was 21.2° ± 7.5°.

**Table 1:** Baseline characteristics of the study cohort (n = 58) IQR: interquartile range

Variable	Value
Mean age at presentation (months)	23.97 ± 13.1
Median age (IQR)	24 (18)
Sex	
Male	29 (50%)
Female	29 (50%)
Unilateral involvement	42 (72.4%)
Bilateral involvement	16 (27.6%)
Presence of Notta’s nodule	54 (93.1%)
Mean initial flexion deformity	21.2° ± 7.5°
Median deformity (IQR)	20° (11°)

Outcome of conservative management

**Figure 2:**
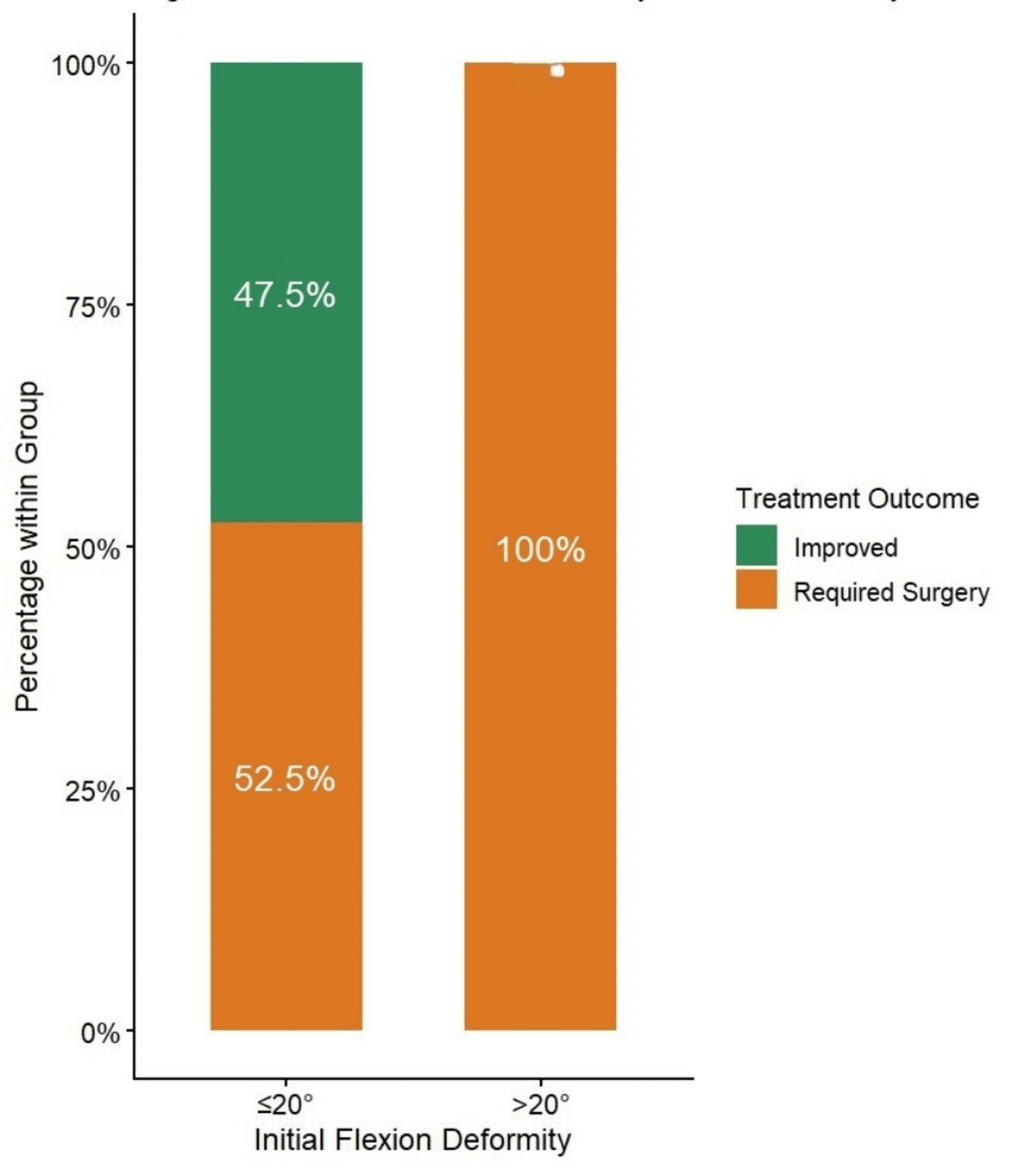
Conservative management outcomes by initial deformity

After six months of observation, 19 of 58 children (32.8%) achieved complete resolution to neutral extension. Improvement was significantly associated with lower initial deformity. Among children with ≤20° deformity, 47.5% improved conservatively, whereas none with >20° deformity improved (Figure 2). The association between initial deformity severity (≤20° vs. >20°) and conservative treatment outcome was analyzed using Pearson’s chi-square test (χ²(1) = 12.41, p = 0.0004, df = 1). Children presenting with an initial flexion deformity greater than 20° had a 1.9-fold higher risk of failure of conservative management than those with a deformity ≤20° (95% CI: 1.32-2.73; p < 0.01).

Surgical outcomes

Thirty-nine children who did not demonstrate adequate improvement after the observation period underwent open A1 pulley release. At six months postoperatively, complete correction (neutral or full active IP extension) was achieved in all cases (39/39; 100%). No recurrence was documented during the six-month postoperative follow-up period. No major complications, including infection, digital nerve injury, stiffness, or wound-related problems, were observed.

**Figure 3:**
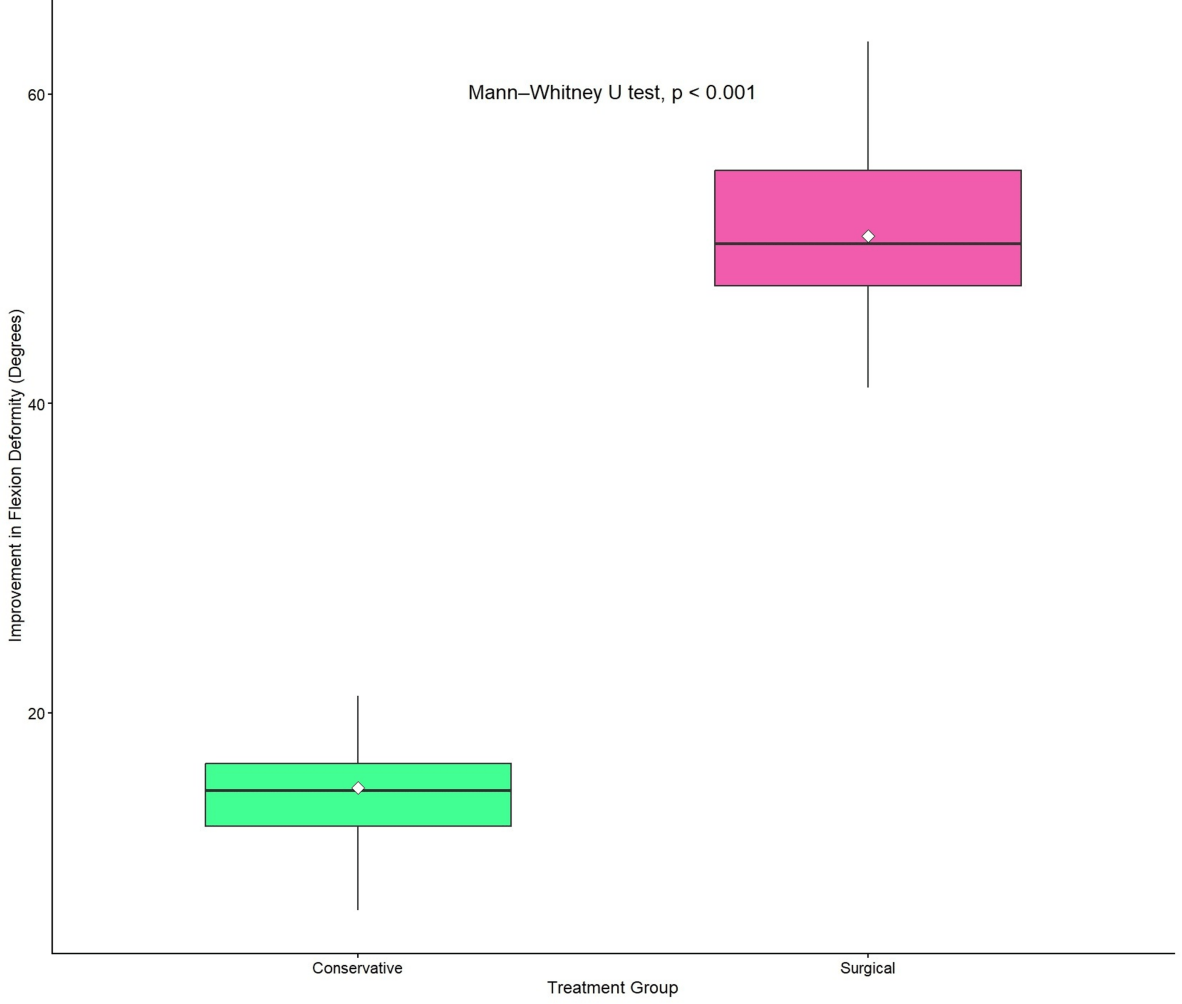
Degree of improvement in flexion deformity

The mean improvement in flexion deformity was significantly greater in the surgical group compared to the conservative group (50.8° ± 5.8° vs. 14.5° ± 3.7°, respectively). Comparison using the Mann-Whitney U test demonstrated a statistically significant difference (U = 42.0; Z = -6.21; p < 0.001) (Figure 3).

Comparative effectiveness analysis

The observed resolution rate was 32.8% in the conservative group, whereas all surgically treated patients achieved complete correction at six-month follow-up. This corresponds to an RR of 3.05 (95% CI: 2.05-4.55), indicating that children who underwent surgical release were approximately three times more likely to achieve resolution than those managed with observation alone; the CI excludes unity, suggesting statistical significance. The ARR was 67.2% (95% CI: 53%-81%), reflecting a substantial absolute difference in resolution rates between the two groups. The calculated NNT was 1.49 (approximately 2), implying that treating two children surgically rather than continuing conservative management would result in one additional case of complete resolution during the follow-up period. However, given the nonrandomized study design and treatment allocation based on response to conservative therapy, these effect estimates should be interpreted as associative rather than causal (Table 2).

**Table 2:** Effect measures (surgery vs. conservative) CI: confidence interval

Measure	Value	95% CI
Relative risk	3.05	2.05-4.55
Absolute risk reduction	67.2%	53%-81%
Number needed to treat	1.49 (~2 patients)	-

Time-to-resolution analysis

The proportional hazard assumption for the Cox regression model was formally evaluated using Schoenfeld residual testing and inspection of log-minus-log survival plots. The Schoenfeld residual test did not demonstrate any statistically significant violation of the proportional hazards assumption (p = 0.34). Visual assessment of the log-minus-log plots also showed approximately parallel survival curves over time. These findings indicate that the proportional hazard assumption was satisfied for the model used in the time-to-resolution analysis.

In this prospective cohort time-to-event analysis, children who underwent surgical intervention had a 4.62-fold higher instantaneous probability of achieving resolution at any given time point than those managed conservatively. The hazard ratio (HR = 4.62) indicates that surgery markedly accelerates recovery. The 95% CI (2.71-7.88) does not cross 1, indicating that this effect is statistically robust and unlikely to be due to chance. The highly significant p value (p < 0.001) further supports the conclusion that surgical treatment results in substantially faster resolution of trigger thumb than conservative management. Clinically, this means that at any point during follow-up, children in the surgical group were more than four times as likely to achieve correction of the deformity as those under observation, indicating a strong time-dependent therapeutic advantage of surgery.

Multivariable logistic regression

Multivariable logistic regression was performed to identify independent predictors of complete resolution. The model included treatment modality, initial deformity >20°, age at presentation, sex, and bilateral involvement. With five predictors and 58 outcome events, the event-per-variable ratio exceeded 10, supporting model stability. Multicollinearity was assessed using variance inflation factors (VIFs; all VIFs <2), indicating no significant collinearity among predictors. Linearity in the logit for the continuous variable (age) was evaluated and showed no significant deviation. Model calibration was adequate (Hosmer-Lemeshow test, p = 0.61), and discrimination was excellent (area under the receiver operating characteristic curve = 0.89). Nagelkerke R² was 0.68. The adjusted odds ratio for initial deformity >20° was derived from the full cohort model, adjusting for treatment modality and other covariates, and therefore reflects an independent prognostic effect rather than an effect limited to the conservative subgroup.

After adjusting for age at presentation, sex, laterality, and initial deformity, surgical intervention remained the strongest independent predictor of successful resolution, with children undergoing surgery having 18.4 times higher odds of complete correction compared with those managed conservatively (95% CI: 5.2-65.3; p < 0.001). This indicates a very strong and statistically significant treatment effect. Initial deformity greater than 20° was independently associated with a markedly lower likelihood of successful outcome under conservative management (adjusted OR = 0.15; 95% CI: 0.05-0.48; p = 0.002). This suggests that children presenting with deformity >20° had approximately 85% lower odds of achieving resolution without surgery, underscoring the prognostic importance of baseline severity. Age at presentation (OR = 1.02; p = 0.41), bilateral involvement (OR = 0.89; p = 0.82), and male sex (OR = 1.11; p = 0.83) were not statistically significant predictors of treatment success, as their CIs crossed unity and p values were >0.05. These findings suggest that demographic factors and laterality did not independently influence outcome after accounting for treatment modality and initial deformity. This model demonstrates that treatment modality and baseline deformity severity are the key determinants of outcome, while age, sex, and bilaterality do not significantly alter the probability of resolution (Table 3).

**Table 3:** Multivariable logistic regression predicting treatment success SE: standard error; OR: odds ratio; CI: confidence interval

Predictor	β	SE	Wald χ²	Adjusted OR	95% CI	p
Surgical treatment	2.91	0.71	16.8	18.4	5.2-65.3	<0.001
Deformity >20°	-1.90	0.60	10.1	0.15	0.05-0.48	0.002
Age	0.02	0.02	0.67	1.02	0.97-1.06	0.41
Male sex	0.10	0.47	0.04	1.11	0.41-3.01	0.83
Bilateral	-0.12	0.53	0.05	0.89	0.32-2.44	0.82

Model diagnostics

The Hosmer-Lemeshow test indicated a p value of 0.61, with a Nagelkerke R² of 0.68 and an AUC of 0.89. No recurrence was observed in either cohort during follow-up. No surgical complications, including infection, nerve injury, stiffness, or wound problems, were recorded. Surgical treatment remained the strongest independent predictor of resolution. An initial deformity of >20° significantly reduced the odds of success. Age, sex, and bilaterality were not significant predictors.

## Discussion

Spontaneous resolution of pediatric trigger thumb has been reported in longitudinal studies, but it typically requires prolonged observation, with mean times to resolution ranging from approximately three to five years, and may occur even later in some cases; however, fixed deformities and more severe contractures are considerably less likely to resolve without surgical intervention. The mean age at presentation (approximately 24 months) aligns with prior studies indicating peak presentation between one and three years of age [1,2]. Bilateral involvement (27.6%) and the high prevalence of Notta’s nodule are consistent with earlier reports [3,5]. The conservative success rate of 32.8% in our study is lower than the 60%-75% spontaneous resolution reported in long-term observational series [6,7]. This difference likely reflects our shorter six-month observation period, as spontaneous recovery may require several years [8]. Baseline deformity severity emerged as a key prognostic factor. None of the children with deformities >20° improved conservatively, supporting previous findings that locked or severe deformities have low rates of spontaneous resolution [8,10]. Surgical release achieved universal correction without recurrence or major complications, consistent with previously published outcomes reporting 95%-100% success [11-14]. The magnitude of deformity correction and accelerated time to resolution underscore the mechanical effectiveness of A1 pulley release. Open release remains preferable to percutaneous techniques in children due to safety concerns regarding neurovascular and tendon injury [15,16]. Multivariable analysis confirmed that treatment modality and baseline severity are the principal determinants of outcome, whereas demographic factors do not independently influence success.

Limitations

Because surgical intervention was offered only to children who did not improve during the observation period, the surgical cohort represents a subset of conservative treatment failures; therefore, baseline comparability between the conservative and surgical groups is inherently limited, and comparative statistics should be interpreted cautiously. Follow-up duration was limited for assessing long-term recurrence. The conservative six-month observation period may underestimate the spontaneous resolution observed in longer term studies. As a single-center study, external validity may be limited. Randomized trials would provide stronger evidence but may pose ethical challenges when delaying definitive treatment.

## Conclusions

This prospective cohort study demonstrates that, although short-term conservative management may result in spontaneous resolution in a subset of mild pediatric trigger thumb cases, open A1 pulley release yields significantly superior and faster outcomes. Surgical intervention achieved universal correction with no recurrence or major complications and was associated with a threefold higher likelihood of resolution compared with conservative management. Initial deformity greater than 20° was the strongest predictor of conservative failure. Early surgical intervention should, therefore, be strongly considered in children presenting with deformity >20°, whereas careful observation may be appropriate for selected mild cases.
